# Ethyl 4-(4-hydroxy­phen­yl)-6-methyl-2-oxo-1,2,3,4-tetra­hydro­pyrimidine-5-carboxyl­ate monohydrate

**DOI:** 10.1107/S1600536808039548

**Published:** 2008-11-29

**Authors:** Ushati Das, Shardul B. Chheda, Suhas R. Pednekar, Narendra P Karambelkar, T. N. Guru Row

**Affiliations:** a Solid State and Structural Chemistry Unit, Indian Institute of Science, Bangalore 560 012, India; b F-12, Organic Chemistry Research Laboratory, Ramanujan Ruia College, Matunga (East), Mumbai 400 019, India; c Jai Research Foundation, C-12, Road No. 16, Wagale Industrial Estate, Thane (West) 400 064, India

## Abstract

There are three formula units in the asymmetric unit of the title compound, C_14_H_16_N_2_O_4_·H_2_O. Mol­ecules are linked by N—H⋯O hydrogen bonds into dimers with the common *R*_2_^2^(8) graph-set motif. Between dimers, single N—H⋯O hydrogen bonds are formed between the other N—H group of each pyrimidine ring and the hydroxyl groups. The water mol­ecules accept O—H⋯O hydrogen bonds from the hydroxyl groups and donate hydrogen bonds to the ester groups.

## Related literature

For background literature concerning pyrimidine compounds and for synthesis details, see: Kappe (2000 ▶); Biginelli (1891 ▶); List (2006 ▶); Mabry & Ganem (2006 ▶).
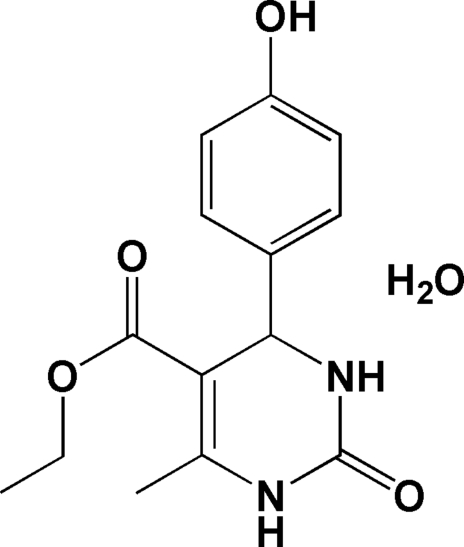


## Experimental

### 

#### Crystal data

C_14_H_16_N_2_O_4_·H_2_O
*M* *_r_* = 294.30
Monoclinic, 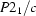

*a* = 11.1583 (15) Å
*b* = 17.773 (2) Å
*c* = 21.686 (3) Å
β = 91.448 (2)°
*V* = 4299.3 (10) Å^3^
*Z* = 12
Mo *K*α radiation
μ = 0.10 mm^−1^
*T* = 292 (2) K
0.50 × 0.20 × 0.10 mm

#### Data collection

Bruker SMART APEX CCD diffractometer
Absorption correction: multi-scan (*SADABS*; Sheldrick, 1996 ▶) *T*_min_ = 0.906, *T*_max_ = 0.990
33204 measured reflections
8419 independent reflections
3947 reflections with *I* > 2σ(*I*)
*R*_int_ = 0.083

#### Refinement

*R*[*F* ^2^ > 2σ(*F* ^2^)] = 0.054
*wR*(*F* ^2^) = 0.138
*S* = 0.90
8419 reflections
601 parameters
H atoms treated by a mixture of independent and constrained refinement
Δρ_max_ = 0.19 e Å^−3^
Δρ_min_ = −0.23 e Å^−3^

### 

Data collection: *SMART* (Bruker, 2004 ▶); cell refinement: *SAINT* (Bruker, 2004 ▶); data reduction: *SAINT*; program(s) used to solve structure: *SHELXS97* (Sheldrick, 2008 ▶); program(s) used to refine structure: *SHELXL97* (Sheldrick, 2008 ▶); molecular graphics: *ORTEP-3 for Windows* (Farrugia, 1999 ▶) and *CAMERON* (Watkin *et al.*, 1993 ▶); software used to prepare material for publication: *PLATON* (Spek, 2003 ▶).

## Supplementary Material

Crystal structure: contains datablocks global, I. DOI: 10.1107/S1600536808039548/bi2319sup1.cif

Structure factors: contains datablocks I. DOI: 10.1107/S1600536808039548/bi2319Isup2.hkl

Additional supplementary materials:  crystallographic information; 3D view; checkCIF report

## Figures and Tables

**Table 1:** Hydrogen-bond geometry (Å, °)

*D*—H⋯*A*	*D*—H	H⋯*A*	*D*⋯*A*	*D*—H⋯*A*
N1—H11⋯O5	0.86	2.14	3.002 (3)	176
N2—H2⋯O2^i^	0.86	2.05	2.882 (3)	162
N3—H3⋯O9	0.86	2.25	3.079 (3)	161
N4—H4⋯O10^ii^	0.86	1.99	2.854 (3)	177
N5—H511⋯O1^iii^	0.86	2.47	3.278 (3)	158
N6—H6⋯O6^iv^	0.86	2.05	2.862 (3)	157
O1—H1⋯O14	0.82	1.84	2.657 (3)	171
O5—H5⋯O13	0.82	1.82	2.633 (3)	172
O9—H9*A*⋯O15	0.82	1.81	2.627 (3)	174
O13—H13*A*⋯O3^v^	0.89 (3)	1.91 (3)	2.789 (3)	172 (3)
O13—H13*B*⋯O2	0.86 (3)	2.02 (3)	2.834 (3)	158 (3)
O14—H14*A*⋯O10^vi^	0.93 (4)	1.88 (4)	2.752 (3)	156 (4)
O14—H14*B*⋯O11^vii^	0.86 (3)	2.02 (3)	2.856 (3)	164 (3)
O15—H15*A*⋯O6	1.03 (4)	1.78 (4)	2.747 (3)	155 (3)
O15—H15*B*⋯O7^viii^	0.88 (3)	1.93 (3)	2.809 (3)	173 (3)

Symmetry codes: (i) 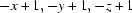; (ii) 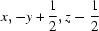; (iii) 
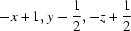
; (iv) 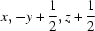; (v) 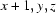; (vi) 
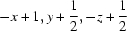
; (vii) 
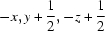
; (viii) 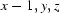.

**Fig. 1.**
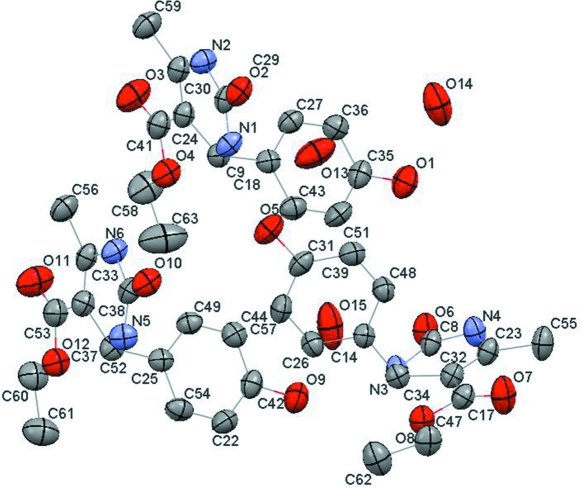
The asymmetric unit of the title compound with displacement ellipsoids drawn at the 50% probability level. H atoms are omitted.

**Fig. 2.**
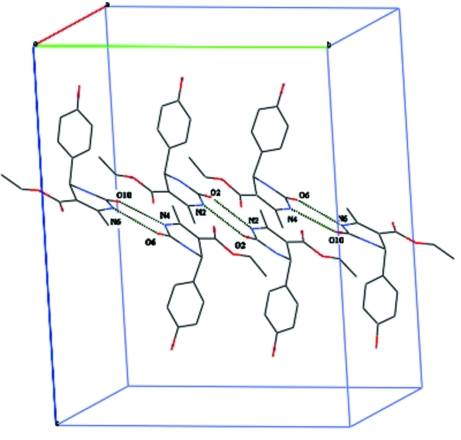
Partial packing diagram showing the R22(8) motifs formed by N—H···O hydrogen bonds.

## supplementary crystallographic information

### Comment

Heterocyclic compounds containing the pyrimidine subunit have been found to have strong bioactivity especially as calcium antagonists. In addition, some of these compounds have also been found to exhibit antihypertensive, antiviral, anti-tumor, antibacterial and anti-inflammatory activity. They can also be potentially used in prevention of coronary artery spasm (Kappe, 2000). The title compound has been synthesized using a Biginelli reaction (Biginelli, 1891), a century-old multi-component reaction (MCR) used to design alkaloids with dihydropyrimidine cores (List, 2006; Mabry & Ganem, 2006).

The compound crystallizes in space group *P*2_1_/*c* with an unusual *Z* value of 12. A search of the Cambridge Structural Database (CSD) yielded only 275 structures having *Z* = 12, which is less than 0.5% of the 75,822 *P*2_1_/*c* structures. In each molecule in the asymmetric unit (Fig. 1), the tetrahydropyrimidine ring adopts a twist boat conformation [C29—N2—C30—C24 = -13.09°, C8—N4—C23—C32 = -14.55°, C38—N6—C33—C37 = 14.90°, C29—N1—C9—C24 = -21.8°, C8—N3—C34—C32 = -31.09°, C38—N5—C52—C37 = 28.07°]. The C—N bond lengths in the tetrahydropyrimidine ring (1.327 (3) to 1.473 (3) Å) are in accordance with those in similar structures. The mean plane of the pyrimidine ring is nearly perpendicular to the phenyl ring [C37—C52—N5 = 110.05°, C14—C34—N3 = 110.25°, C18—C9—N1 = 110.38°].

The structure contains extensive intermolecular N—H···O hydrogen bonding (see Table). Atom N2 acts as a hydrogen-bond donor to O2 to form a centrosymmetric dimer centered at (1/2,1/2,1/2) with the common *R*_2_^2^(8) graph-set motif. Atom N6 acts as a hydrogen-bond donor to O6 and N4 acts as a donor to O10 to form another dimeric *R*_2_^2^(8) motif. However, such *R*_2_^2^(8) motifs are not generated *via* N1, N3 and N5. Instead, these N atoms form single N—H···O hydrogen bonds to hydroxyl groups: N1 to O5, N3 to O9 and N5 to O1.

### Experimental

4-Hydroxybenzaldehyde (0.01 mol), ethyl acetoacetate (0.01 mol), urea (0.02 mol) and *p*-TSA (0.002 mol) were ground for 4-5 min using a mortar and pestle. The initial syrupy reaction mixture solidified within 15 min. The solid was filtered, washed and recrystallized from a water-acetic acid mixture (yield 95%, m.p. 509–511 K).

^1^H NMR (DMSO):δ 6.66 (*d*, 2H), 7.02 (*d*, 2H), 1.07 (*t*, 2H), 3.98 (*q*, 2 H), 2.23 (*s*, 3 H), 5.08 (*s*, 1 H), 7.58 (*s*, 1H), 9.08 (*s*, 1H), 9.30 (*s*, 1H).

### Refinement

H atoms bound to C atoms were placed geometrically and allowed to ride during subsequent refinement with C—H = 0.93 (3)–0.98 (3) Å and *U*_iso_(H) = 1.2 or 1.5 *U*_eq_(C). H atoms of the hydroxyl groups were placed geometrically with C—H = 0.82 Å and allowed to rotate around the C—O bond with *U*_iso_(H) = 1.5 *U*_eq_(O). H atoms of the water molecules were located in difference Fourier maps and refined freely with isotropic displacement parameters.

### Crystal data

**Table:**

C_14_H_16_N_2_O_4_·H_2_O	*F*_000_ = 1872
*M**_r_* = 294.30	*D*_x_ = 1.364 Mg m^−^^3^
Monoclinic, *P*2_1_/*c*	Mo *K*α radiation λ = 0.71073 Å
Hall symbol: -P 2ybc	Cell parameters from 1328 reflections
*a* = 11.1583 (15) Å	θ = 1.8–26.0º
*b* = 17.773 (2) Å	µ = 0.10 mm^−^^1^
*c* = 21.686 (3) Å	*T* = 292 (2) K
β = 91.448 (2)º	Lath, colourless
*V* = 4299.3 (10) Å^3^	0.50 × 0.20 × 0.10 mm
*Z* = 12	

### Data collection

**Table:**

Bruker SMART APEX CCD diffractometer	8419 independent reflections
Radiation source: fine-focus sealed tube	3947 reflections with *I* > 2σ(*I*)
Monochromator: graphite	*R*_int_ = 0.083
*T* = 292(2) K	θ_max_ = 26.0º
φ and ω scans	θ_min_ = 1.8º
Absorption correction: multi-scan(SADABS; Sheldrick, 1996)	*h* = −13→13
*T*_min_ = 0.906, *T*_max_ = 0.990	*k* = −20→21
33204 measured reflections	*l* = −26→26

### Refinement

**Table:**

Refinement on *F*^2^	Secondary atom site location: difference Fourier map
Least-squares matrix: full	Hydrogen site location: inferred from neighbouring sites
*R*[*F*^2^ > 2σ(*F*^2^)] = 0.054	H atoms treated by a mixture of independent and constrained refinement
*wR*(*F*^2^) = 0.138	*w* = 1/[σ^2^(*F*_o_^2^) + (0.0537*P*)^2^ + 0.2183*P*] where *P* = (*F*_o_^2^ + 2*F*_c_^2^)/3
*S* = 0.90	(Δ/σ)_max_ = 0.048
8419 reflections	Δρ_max_ = 0.19 e Å^−^^3^
601 parameters	Δρ_min_ = −0.23 e Å^−^^3^
Primary atom site location: structure-invariant direct methods	Extinction correction: none

### Special details

**Table:**

Geometry. Bond distances, angles *etc*. have been calculated using the rounded fractional coordinates. All su's are estimated from the variances of the (full) variance-covariance matrix. The cell e.s.d.'s are taken into account in the estimation of distances, angles and torsion angles
Refinement. Refinement of *F*^2^ against ALL reflections. The weighted *R*-factor *wR* and goodness of fit *S* are based on *F*^2^, conventional *R*-factors *R* are based on *F*, with *F* set to zero for negative *F*^2^. The threshold expression of *F*^2^ > σ(*F*^2^) is used only for calculating *R*-factors(gt) *etc*. and is not relevant to the choice of reflections for refinement. *R*-factors based on *F*^2^ are statistically about twice as large as those based on *F*, and *R*- factors based on ALL data will be even larger.

### Fractional atomic coordinates and isotropic or equivalent isotropic displacement parameters (Å^2^)

**Table:**

	*x*	*y*	*z*	*U*_iso_*/*U*_eq_	
O1	0.33773 (18)	0.41538 (12)	0.11596 (8)	0.0621 (8)	
O2	0.57587 (15)	0.42885 (10)	0.45666 (8)	0.0497 (7)	
O3	0.02224 (16)	0.37128 (12)	0.41500 (9)	0.0719 (9)	
O4	0.11134 (15)	0.28500 (11)	0.35739 (8)	0.0557 (7)	
N1	0.45115 (16)	0.35440 (12)	0.39975 (9)	0.0422 (8)	
N2	0.37628 (18)	0.45170 (12)	0.45626 (9)	0.0458 (8)	
C9	0.3345 (2)	0.33601 (14)	0.37022 (11)	0.0389 (9)	
C18	0.3331 (2)	0.35869 (14)	0.30237 (11)	0.0367 (9)	
C24	0.2357 (2)	0.37138 (15)	0.40681 (11)	0.0391 (9)	
C27	0.2886 (2)	0.42710 (15)	0.28185 (12)	0.0425 (10)	
C29	0.4734 (2)	0.41183 (15)	0.43768 (11)	0.0400 (10)	
C30	0.2591 (2)	0.42843 (15)	0.44647 (11)	0.0407 (10)	
C35	0.3341 (2)	0.39763 (17)	0.17742 (12)	0.0452 (10)	
C36	0.2889 (2)	0.44681 (15)	0.22048 (12)	0.0447 (10)	
C41	0.1130 (2)	0.34506 (17)	0.39467 (12)	0.0469 (10)	
C43	0.3778 (2)	0.31030 (15)	0.25840 (12)	0.0461 (10)	
C51	0.3789 (2)	0.32967 (16)	0.19670 (12)	0.0511 (11)	
C58	−0.0060 (2)	0.25637 (19)	0.33819 (14)	0.0726 (13)	
C59	0.1717 (2)	0.47050 (15)	0.48439 (12)	0.0564 (11)	
C63	0.0100 (3)	0.19780 (19)	0.29252 (17)	0.0982 (16)	
O5	0.66400 (17)	0.25970 (11)	0.36851 (8)	0.0604 (8)	
O6	0.43755 (14)	0.22711 (11)	0.03821 (8)	0.0521 (7)	
O7	0.99348 (15)	0.17389 (11)	0.07701 (9)	0.0656 (8)	
O8	0.90818 (14)	0.09252 (10)	0.14113 (8)	0.0464 (7)	
N3	0.56592 (16)	0.15289 (12)	0.09325 (9)	0.0402 (8)	
N4	0.63647 (17)	0.25030 (12)	0.03655 (9)	0.0458 (8)	
C8	0.5406 (2)	0.21040 (15)	0.05610 (11)	0.0392 (10)	
C14	0.67912 (19)	0.17523 (14)	0.19033 (11)	0.0340 (9)	
C17	0.9035 (2)	0.14900 (16)	0.09962 (12)	0.0424 (10)	
C23	0.7551 (2)	0.22858 (15)	0.04565 (11)	0.0410 (10)	
C26	0.6355 (2)	0.13308 (15)	0.23857 (11)	0.0434 (10)	
C31	0.6696 (2)	0.23365 (16)	0.30919 (12)	0.0426 (10)	
C32	0.7804 (2)	0.17388 (14)	0.08680 (11)	0.0366 (9)	
C34	0.6820 (2)	0.14156 (14)	0.12561 (10)	0.0368 (9)	
C39	0.7103 (2)	0.27746 (15)	0.26171 (12)	0.0451 (10)	
C47	1.0265 (2)	0.06549 (16)	0.15924 (12)	0.0519 (10)	
C48	0.7151 (2)	0.24819 (14)	0.20283 (11)	0.0416 (10)	
C55	0.8393 (2)	0.27145 (16)	0.00655 (11)	0.0544 (10)	
C57	0.6307 (2)	0.16154 (16)	0.29722 (12)	0.0484 (11)	
C62	1.0128 (3)	0.01022 (16)	0.21043 (14)	0.0651 (11)	
O9	0.35723 (18)	0.05785 (11)	0.14122 (8)	0.0603 (8)	
O10	0.58206 (15)	0.11471 (10)	0.47033 (8)	0.0522 (7)	
O11	0.02324 (17)	0.06082 (12)	0.43805 (10)	0.0759 (9)	
O12	0.10976 (14)	−0.03730 (11)	0.39298 (8)	0.0509 (7)	
N5	0.45147 (17)	0.03034 (12)	0.42832 (9)	0.0443 (8)	
N6	0.38344 (17)	0.14147 (12)	0.46649 (9)	0.0440 (8)	
C22	0.3837 (2)	−0.01916 (16)	0.22853 (13)	0.0488 (11)	
C25	0.3366 (2)	0.02517 (15)	0.33034 (11)	0.0369 (9)	
C33	0.2636 (2)	0.11928 (15)	0.46115 (11)	0.0411 (10)	
C37	0.2370 (2)	0.05563 (15)	0.43094 (11)	0.0406 (9)	
C38	0.4785 (2)	0.09551 (16)	0.45504 (11)	0.0410 (10)	
C42	0.3491 (2)	0.04858 (17)	0.20369 (12)	0.0449 (10)	
C44	0.3102 (2)	0.10538 (15)	0.24164 (12)	0.0442 (10)	
C49	0.3046 (2)	0.09334 (15)	0.30425 (12)	0.0420 (10)	
C52	0.3344 (2)	0.01124 (15)	0.39965 (11)	0.0386 (9)	
C53	0.1136 (2)	0.02892 (17)	0.42254 (12)	0.0460 (10)	
C54	0.3758 (2)	−0.03085 (15)	0.29138 (13)	0.0449 (10)	
C56	0.1815 (2)	0.17336 (15)	0.49137 (12)	0.0544 (11)	
C60	−0.0077 (2)	−0.06677 (15)	0.37706 (13)	0.0553 (11)	
C61	0.0072 (3)	−0.13722 (16)	0.34069 (14)	0.0699 (14)	
O13	0.7780 (2)	0.38763 (15)	0.38773 (12)	0.0812 (10)	
O14	0.2287 (2)	0.54269 (15)	0.08331 (12)	0.0840 (11)	
O15	0.2419 (2)	0.17780 (15)	0.10108 (11)	0.0879 (10)	
H1	0.30135	0.45482	0.10960	0.0932*	
H2	0.38863	0.49366	0.47513	0.0549*	
H9	0.32437	0.28129	0.37210	0.0467*	
H11	0.51035	0.32531	0.39174	0.0506*	
H27	0.25777	0.46068	0.31026	0.0509*	
H36	0.25859	0.49325	0.20793	0.0537*	
H43	0.40775	0.26369	0.27072	0.0553*	
H51	0.41020	0.29641	0.16817	0.0612*	
H58A	−0.04686	0.23614	0.37349	0.0871*	
H58B	−0.05442	0.29676	0.32070	0.0871*	
H59A	0.09680	0.47487	0.46189	0.0845*	
H59B	0.20257	0.51979	0.49344	0.0845*	
H59C	0.15941	0.44387	0.52223	0.0845*	
H63A	0.05611	0.21716	0.25926	0.1474*	
H63B	−0.06690	0.18151	0.27685	0.1474*	
H63C	0.05152	0.15599	0.31123	0.1474*	
H3	0.51070	0.11999	0.09877	0.0483*	
H4	0.62266	0.29180	0.01724	0.0550*	
H5	0.70400	0.29823	0.37209	0.0906*	
H26	0.60883	0.08429	0.23100	0.0521*	
H34	0.69565	0.08730	0.12966	0.0441*	
H39	0.73457	0.32672	0.26929	0.0541*	
H47A	1.07654	0.10717	0.17294	0.0624*	
H47B	1.06404	0.04155	0.12448	0.0624*	
H48	0.74299	0.27808	0.17104	0.0499*	
H55A	0.85834	0.31867	0.02594	0.0813*	
H55B	0.80233	0.28044	−0.03322	0.0813*	
H55C	0.91146	0.24286	0.00175	0.0813*	
H57	0.60112	0.13213	0.32889	0.0581*	
H62A	0.96886	0.03302	0.24302	0.0977*	
H62B	1.09059	−0.00470	0.22590	0.0977*	
H62C	0.97019	−0.03319	0.19528	0.0977*	
H6	0.39839	0.18699	0.47771	0.0527*	
H9A	0.31708	0.09432	0.13021	0.0904*	
H22	0.41239	−0.05706	0.20328	0.0583*	
H44	0.28789	0.15168	0.22501	0.0530*	
H49	0.27868	0.13203	0.32952	0.0503*	
H52	0.31950	−0.04243	0.40642	0.0464*	
H54	0.39733	−0.07741	0.30781	0.0538*	
H56A	0.19101	0.16920	0.53535	0.0815*	
H56B	0.20054	0.22368	0.47884	0.0815*	
H56C	0.10009	0.16203	0.47936	0.0815*	
H60A	−0.05117	−0.07733	0.41424	0.0662*	
H60B	−0.05286	−0.03009	0.35289	0.0662*	
H61A	0.04801	−0.17420	0.36572	0.1045*	
H61B	−0.07023	−0.15608	0.32804	0.1045*	
H61C	0.05328	−0.12674	0.30491	0.1045*	
H511	0.50667	−0.00340	0.42770	0.0532*	
H13A	0.854 (3)	0.3808 (19)	0.4000 (15)	0.109 (14)*	
H13B	0.730 (3)	0.409 (2)	0.4129 (16)	0.117 (15)*	
H14A	0.276 (4)	0.570 (2)	0.0565 (18)	0.140 (17)*	
H14B	0.157 (3)	0.555 (2)	0.0722 (17)	0.121 (16)*	
H15A	0.297 (4)	0.199 (2)	0.0682 (17)	0.153 (16)*	
H15B	0.165 (3)	0.1797 (18)	0.0914 (14)	0.096 (13)*	

### Atomic displacement parameters (Å^2^)

**Table:**

	*U*^11^	*U*^22^	*U*^33^	*U*^12^	*U*^13^	*U*^23^
O1	0.0634 (14)	0.0856 (17)	0.0372 (12)	0.0017 (12)	−0.0015 (10)	0.0022 (10)
O2	0.0299 (10)	0.0717 (14)	0.0473 (12)	−0.0014 (10)	−0.0008 (9)	−0.0075 (10)
O3	0.0325 (12)	0.1028 (18)	0.0806 (16)	−0.0027 (11)	0.0056 (10)	−0.0294 (13)
O4	0.0359 (11)	0.0668 (14)	0.0643 (13)	−0.0062 (10)	0.0002 (9)	−0.0132 (11)
N1	0.0281 (12)	0.0581 (16)	0.0401 (13)	0.0072 (11)	−0.0022 (10)	−0.0083 (12)
N2	0.0324 (13)	0.0503 (15)	0.0547 (15)	0.0007 (11)	0.0023 (11)	−0.0085 (12)
C9	0.0346 (15)	0.0408 (17)	0.0413 (16)	−0.0010 (12)	0.0000 (12)	−0.0005 (13)
C18	0.0279 (13)	0.0398 (17)	0.0422 (16)	−0.0020 (12)	−0.0035 (12)	−0.0039 (13)
C24	0.0309 (14)	0.0492 (18)	0.0371 (16)	−0.0001 (13)	0.0020 (12)	0.0023 (14)
C27	0.0368 (15)	0.0492 (19)	0.0414 (18)	0.0023 (13)	0.0011 (13)	−0.0066 (14)
C29	0.0313 (16)	0.0551 (19)	0.0335 (16)	−0.0006 (14)	0.0015 (12)	0.0042 (14)
C30	0.0316 (15)	0.0512 (19)	0.0392 (16)	0.0011 (13)	0.0010 (12)	0.0024 (14)
C35	0.0376 (16)	0.062 (2)	0.0359 (17)	−0.0069 (14)	−0.0004 (13)	−0.0024 (15)
C36	0.0392 (16)	0.0460 (18)	0.0489 (18)	0.0012 (13)	−0.0010 (13)	0.0004 (14)
C41	0.0368 (16)	0.061 (2)	0.0431 (18)	0.0006 (15)	0.0027 (13)	0.0029 (15)
C43	0.0395 (16)	0.0473 (18)	0.0514 (19)	0.0042 (13)	−0.0003 (14)	−0.0063 (14)
C51	0.0518 (18)	0.060 (2)	0.0418 (18)	0.0059 (16)	0.0056 (14)	−0.0144 (16)
C58	0.0372 (17)	0.096 (3)	0.084 (2)	−0.0118 (17)	−0.0087 (16)	−0.027 (2)
C59	0.0444 (17)	0.069 (2)	0.056 (2)	0.0030 (16)	0.0080 (14)	−0.0133 (16)
C63	0.057 (2)	0.097 (3)	0.139 (3)	0.011 (2)	−0.026 (2)	−0.054 (3)
O5	0.0576 (14)	0.0820 (17)	0.0417 (12)	0.0082 (11)	0.0026 (10)	−0.0114 (11)
O6	0.0270 (10)	0.0748 (14)	0.0543 (12)	0.0008 (10)	−0.0032 (9)	0.0147 (10)
O7	0.0294 (11)	0.0941 (16)	0.0734 (14)	0.0036 (10)	0.0048 (10)	0.0304 (12)
O8	0.0317 (10)	0.0578 (13)	0.0496 (12)	0.0032 (9)	−0.0022 (8)	0.0079 (10)
N3	0.0318 (12)	0.0496 (15)	0.0391 (13)	−0.0108 (10)	−0.0032 (10)	0.0063 (11)
N4	0.0306 (12)	0.0542 (15)	0.0527 (14)	0.0001 (11)	0.0004 (10)	0.0168 (12)
C8	0.0321 (15)	0.0518 (19)	0.0338 (16)	−0.0057 (14)	0.0033 (12)	0.0001 (14)
C14	0.0258 (13)	0.0406 (17)	0.0355 (15)	−0.0023 (12)	−0.0015 (11)	0.0010 (13)
C17	0.0365 (16)	0.0526 (19)	0.0381 (16)	0.0017 (14)	0.0009 (13)	−0.0011 (14)
C23	0.0287 (14)	0.0584 (19)	0.0359 (16)	0.0008 (13)	0.0024 (12)	0.0008 (14)
C26	0.0441 (16)	0.0421 (17)	0.0440 (18)	−0.0064 (13)	0.0001 (13)	0.0040 (14)
C31	0.0345 (15)	0.058 (2)	0.0352 (16)	0.0096 (14)	0.0010 (12)	−0.0061 (15)
C32	0.0282 (14)	0.0474 (18)	0.0341 (15)	0.0012 (12)	−0.0030 (11)	−0.0003 (13)
C34	0.0291 (14)	0.0411 (16)	0.0399 (16)	0.0011 (12)	−0.0046 (12)	0.0035 (13)
C39	0.0403 (16)	0.0453 (18)	0.0495 (19)	0.0000 (13)	−0.0004 (13)	−0.0054 (15)
C47	0.0318 (15)	0.067 (2)	0.0568 (19)	0.0066 (14)	−0.0026 (13)	0.0089 (16)
C48	0.0437 (16)	0.0440 (18)	0.0370 (16)	−0.0037 (13)	0.0002 (12)	0.0037 (14)
C55	0.0383 (16)	0.080 (2)	0.0450 (18)	−0.0029 (15)	0.0054 (13)	0.0156 (15)
C57	0.0512 (18)	0.059 (2)	0.0354 (17)	−0.0001 (15)	0.0069 (13)	0.0072 (15)
C62	0.0480 (19)	0.070 (2)	0.077 (2)	0.0000 (16)	−0.0039 (16)	0.0214 (18)
O9	0.0626 (14)	0.0743 (16)	0.0444 (12)	−0.0070 (11)	0.0098 (10)	−0.0001 (10)
O10	0.0288 (10)	0.0685 (14)	0.0592 (13)	−0.0029 (9)	−0.0033 (9)	−0.0138 (10)
O11	0.0315 (12)	0.0871 (17)	0.1094 (18)	−0.0028 (11)	0.0055 (11)	−0.0312 (13)
O12	0.0302 (10)	0.0586 (14)	0.0637 (13)	−0.0065 (9)	−0.0012 (9)	−0.0062 (11)
N5	0.0310 (12)	0.0509 (16)	0.0505 (14)	0.0057 (11)	−0.0108 (10)	−0.0023 (12)
N6	0.0308 (12)	0.0519 (15)	0.0492 (14)	−0.0020 (11)	0.0015 (10)	−0.0118 (11)
C22	0.0477 (18)	0.051 (2)	0.0478 (19)	0.0030 (15)	0.0062 (14)	−0.0099 (15)
C25	0.0276 (14)	0.0390 (17)	0.0437 (17)	−0.0014 (12)	−0.0043 (12)	−0.0044 (14)
C33	0.0301 (14)	0.059 (2)	0.0342 (15)	0.0027 (14)	0.0019 (12)	0.0018 (14)
C37	0.0313 (15)	0.0489 (18)	0.0414 (16)	−0.0010 (13)	−0.0003 (12)	−0.0002 (14)
C38	0.0361 (16)	0.053 (2)	0.0339 (16)	0.0029 (14)	−0.0011 (12)	0.0003 (14)
C42	0.0357 (16)	0.061 (2)	0.0380 (17)	−0.0056 (14)	0.0025 (13)	0.0009 (16)
C44	0.0403 (16)	0.0462 (18)	0.0459 (18)	−0.0027 (13)	−0.0023 (13)	0.0001 (15)
C49	0.0413 (16)	0.0430 (18)	0.0413 (17)	0.0030 (13)	−0.0036 (12)	−0.0055 (14)
C52	0.0264 (14)	0.0466 (18)	0.0427 (16)	−0.0025 (12)	−0.0035 (12)	−0.0023 (13)
C53	0.0349 (16)	0.055 (2)	0.0481 (18)	−0.0014 (15)	−0.0001 (13)	0.0033 (15)
C54	0.0357 (15)	0.0398 (17)	0.059 (2)	0.0057 (13)	−0.0021 (13)	−0.0015 (15)
C56	0.0411 (16)	0.068 (2)	0.0544 (19)	0.0036 (15)	0.0084 (14)	−0.0106 (15)
C60	0.0366 (16)	0.059 (2)	0.070 (2)	−0.0095 (14)	−0.0065 (14)	−0.0077 (16)
C61	0.056 (2)	0.060 (2)	0.093 (3)	0.0000 (17)	−0.0132 (18)	−0.0122 (19)
O13	0.0445 (15)	0.109 (2)	0.0898 (18)	0.0123 (14)	−0.0036 (14)	−0.0457 (15)
O14	0.0466 (15)	0.102 (2)	0.103 (2)	−0.0050 (14)	−0.0046 (14)	0.0502 (16)
O15	0.0438 (14)	0.131 (2)	0.0886 (19)	−0.0107 (15)	−0.0047 (13)	0.0552 (16)

### Geometric parameters (Å, °)

**Table:**

O1—C35	1.371 (3)	C58—H58B	0.97
O2—C29	1.243 (3)	C58—H58A	0.97
O3—C41	1.208 (3)	C59—H59A	0.96
O4—C41	1.339 (3)	C59—H59C	0.96
O4—C58	1.456 (3)	C59—H59B	0.96
O1—H1	0.82	C63—H63B	0.96
O5—C31	1.370 (3)	C63—H63C	0.96
O6—C8	1.240 (3)	C63—H63A	0.96
O7—C17	1.212 (3)	C14—C34	1.527 (3)
O8—C17	1.349 (3)	C14—C26	1.385 (3)
O8—C47	1.450 (3)	C14—C48	1.382 (3)
O5—H5	0.82	C17—C32	1.463 (3)
O9—C42	1.370 (3)	C23—C55	1.491 (3)
O10—C38	1.242 (3)	C23—C32	1.345 (4)
O11—C53	1.212 (3)	C26—C57	1.371 (4)
O12—C60	1.445 (3)	C31—C57	1.376 (4)
O12—C53	1.340 (4)	C31—C39	1.377 (4)
O9—H9A	0.82	C32—C34	1.514 (3)
O13—H13A	0.89 (3)	C39—C48	1.381 (4)
O13—H13B	0.86 (3)	C47—C62	1.493 (4)
O14—H14A	0.93 (4)	C26—H26	0.93
O14—H14B	0.86 (3)	C34—H34	0.98
N1—C29	1.330 (3)	C39—H39	0.93
N1—C9	1.473 (3)	C47—H47B	0.97
N2—C29	1.364 (3)	C47—H47A	0.97
N2—C30	1.383 (3)	C48—H48	0.93
N1—H11	0.86	C55—H55C	0.96
N2—H2	0.86	C55—H55A	0.96
O15—H15B	0.88 (3)	C55—H55B	0.96
O15—H15A	1.03 (4)	C57—H57	0.93
N3—C34	1.471 (3)	C62—H62C	0.96
N3—C8	1.327 (3)	C62—H62B	0.96
N4—C23	1.388 (3)	C62—H62A	0.96
N4—C8	1.360 (3)	C22—C54	1.384 (4)
N3—H3	0.86	C22—C42	1.371 (4)
N4—H4	0.86	C25—C54	1.384 (4)
N5—C52	1.472 (3)	C25—C52	1.524 (3)
N5—C38	1.327 (3)	C25—C49	1.380 (4)
N6—C38	1.367 (3)	C33—C56	1.491 (4)
N6—C33	1.396 (3)	C33—C37	1.337 (4)
N5—H511	0.86	C37—C53	1.464 (3)
N6—H6	0.86	C37—C52	1.517 (3)
C9—C24	1.511 (3)	C42—C44	1.379 (4)
C9—C18	1.525 (3)	C44—C49	1.378 (4)
C18—C27	1.383 (4)	C60—C61	1.491 (4)
C18—C43	1.386 (4)	C22—H22	0.93
C24—C30	1.351 (4)	C44—H44	0.93
C24—C41	1.464 (3)	C49—H49	0.93
C27—C36	1.376 (4)	C52—H52	0.98
C30—C59	1.492 (3)	C54—H54	0.93
C35—C36	1.384 (4)	C56—H56A	0.96
C35—C51	1.369 (4)	C56—H56B	0.96
C43—C51	1.382 (4)	C56—H56C	0.96
C58—C63	1.451 (5)	C60—H60A	0.97
C9—H9	0.98	C60—H60B	0.97
C27—H27	0.93	C61—H61C	0.96
C36—H36	0.93	C61—H61A	0.96
C43—H43	0.93	C61—H61B	0.96
C51—H51	0.93		
			
O1···O14	2.657 (3)	C51···H54^ii^	3.00
O2···C8^i^	3.313 (3)	C53···H14B^viii^	3.06 (3)
O2···O13	2.834 (3)	C53···H56C	2.67
O2···O9^ii^	3.225 (3)	C54···H39^vii^	3.09
O2···N2^iii^	2.882 (3)	C56···H60A^xi^	3.06
O3···C59	2.834 (3)	C56···H15A^i^	3.08 (4)
O3···O13^iv^	2.789 (3)	C58···H13A^iv^	3.04 (3)
O4···C18	3.068 (3)	C60···H14B^viii^	2.96 (4)
O5···O13	2.633 (3)	H1···H14A	2.36
O5···N1	32 (3)	H1···H36	2.30
O6···N6^v^	2.862 (3)	H1···H511^ii^	2.43
O6···O15	2.747 (3)	H1···H14B	2.52
O6···C29^v^	3.325 (3)	H1···O14	1.84
O7···C55	2.858 (3)	H2···O2^iii^	2.05
O7···O15^vi^	2.809 (3)	H2···H59B	2.17
O8···C14	3.158 (3)	H2···C29^iii^	2.94
O9···N3	3.079 (3)	H3···O9	2.25
O9···O2^vii^	3.225 (3)	H3···H9A	2.33
O9···O15	2.627 (3)	H4···O10^v^	1.99
O10···O14^vii^	2.752 (3)	H4···H55B	2.32
O10···N4^i^	2.854 (3)	H4···C38^v^	2.88
O11···O14^viii^	2.856 (3)	H5···H13B	2.17
O11···C56	2.890 (3)	H5···H13A	2.30
O12···C25	3.108 (3)	H5···O13	1.82
O13···O5	2.633 (3)	H5···H39	2.32
O13···O3^vi^	2.789 (3)	H5···H11	2.26
O13···O2	2.834 (3)	H6···O6^i^	2.05
O14···O11^ix^	2.856 (3)	H6···H56B	2.30
O14···C60^ix^	3.273 (3)	H6···C8^i^	2.93
O14···O10^ii^	2.752 (3)	H9···O4	2.39
O14···O1	2.657 (3)	H9···H43	2.43
O15···C42	3.395 (4)	H9A···H44	2.33
O15···O9	2.627 (3)	H9A···H15A	2.30
O15···C47^iv^	3.392 (4)	H9A···H15B	2.41
O15···C44	3.378 (4)	H9A···H3	2.33
O15···O6	2.747 (3)	H9A···O15	1.81
O15···O7^iv^	2.809 (3)	H11···C31	3.03
O1···H511^ii^	2.47	H11···H5	2.26
O1···H56A^v^	2.80	H11···O5	2.14
O2···H13B	2.02 (3)	H13A···O3^vi^	1.91 (3)
O2···H2^iii^	2.05	H13A···H5	2.30
O2···H59B^iii^	2.83	H13A···C58^vi^	3.04 (3)
O3···H13A^iv^	1.91 (3)	H13A···C41^vi^	2.96 (3)
O3···H58B	2.57	H13A···H58B^vi^	2.51
O3···H58A	2.67	H13B···O5	2.91 (4)
O3···H59A	2.25	H13B···H5	2.17
O4···H9	2.39	H13B···O2	2.02 (3)
O5···H11	2.14	H13B···H59B^iii^	2.49
O5···H13B	2.91 (4)	H13B···C29	2.93 (3)
O5···H55B^i^	2.70	H14A···C38^ii^	2.79 (4)
O6···H15A	1.78 (4)	H14A···O10^ii^	1.88 (4)
O6···H6^v^	2.05	H14A···H1	2.36
O7···H15B^vi^	1.93 (3)	H14B···H1	2.52
O7···H55C	2.22	H14B···C60^ix^	2.96 (4)
O7···H47A	2.55	H14B···C53^ix^	3.06 (3)
O7···H47B	2.68	H14B···O11^ix^	2.02 (3)
O8···H34	2.38	H14B···H60B^ix^	2.52
O9···H3	2.25	H15A···C8	2.75 (4)
O10···H4^i^	1.99	H15A···C56^v^	3.08 (4)
O10···H14A^vii^	1.88 (4)	H15A···H9A	2.30
O11···H60B	2.58	H15A···O6	1.78 (4)
O11···H14B^viii^	2.02 (3)	H15A···H56B^v^	2.59
O11···H60A	2.64	H15B···O7^iv^	1.93 (3)
O11···H56C	2.18	H15B···C17^iv^	2.98 (3)
O12···H52	2.35	H15B···C47^iv^	2.96 (3)
O13···H58B^vi^	2.89	H15B···H9A	2.41
O13···H5	1.82	H15B···H47A^iv^	2.42
O13···H39	2.82	H26···C42	3.01
O14···H60A^ix^	2.91	H26···C36^vii^	2.88
O14···H36	2.85	H26···H34	2.42
O14···H1	1.84	H26···C27^vii^	3.03
O14···H60B^ix^	2.75	H26···H36^vii^	2.54
O15···H59C^v^	2.89	H27···H62C^ii^	2.55
O15···H47A^iv^	2.75	H27···C24	2.64
O15···H9A	1.81	H27···C30	3.01
O15···H44	2.76	H34···H26	2.42
N1···O5	32 (3)	H34···O8	2.38
N2···O2^iii^	2.882 (3)	H36···O14	2.85
N3···O9	3.079 (3)	H36···H26^ii^	2.54
N4···O10^v^	2.854 (3)	H36···H1	2.30
N6···O6^i^	2.862 (3)	H36···C26^ii^	2.97
C8···O2^v^	3.313 (3)	H39···C54^ii^	3.09
C8···C29^v^	3.432 (4)	H39···O13	2.82
C14···O8	3.158 (3)	H39···C22^ii^	3.04
C17···C48	3.574 (3)	H39···H5	2.32
C18···O4	3.068 (3)	H43···H44	2.58
C23···C48	3.466 (3)	H43···H9	2.43
C25···O12	3.108 (3)	H43···C44	3.08
C26···C36^vii^	3.525 (4)	H43···C31	3.07
C26···C42	3.594 (3)	H44···C43	3.07
C27···C30	3.593 (4)	H44···O15	2.76
C27···C41	3.492 (4)	H44···H9A	2.33
C29···O6^i^	3.325 (3)	H44···H43	2.58
C29···C8^i^	3.432 (4)	H47A···H15B^vi^	2.42
C30···C27	3.593 (4)	H47A···C44^vi^	2.97
C33···C49	3.475 (4)	H47A···O7	2.55
C35···C54^ii^	3.527 (3)	H47A···O15^vi^	2.75
C36···C26^ii^	3.525 (4)	H47B···O7	2.68
C39···C58^vi^	3.556 (3)	H47B···H59C^xii^	2.50
C41···C27	3.492 (4)	H48···C32	2.64
C42···O15	3.395 (4)	H48···C23	2.86
C42···C26	3.594 (3)	H49···H63C	2.59
C44···O15	3.378 (4)	H49···C37	2.64
C47···O15^vi^	3.392 (4)	H49···C33	2.87
C48···C23	3.466 (3)	H52···H54	2.41
C48···C17	3.574 (3)	H52···O12	2.35
C49···C33	3.475 (4)	H54···C35^vii^	3.04
C49···C53	3.565 (4)	H54···C51^vii^	3.00
C53···C49	3.565 (4)	H54···H52	2.41
C54···C35^vii^	3.527 (3)	H55A···H61A^ii^	2.55
C55···O7	2.858 (3)	H55B···O5^v^	2.70
C56···O11	2.890 (3)	H55B···H4	2.32
C58···C39^iv^	3.556 (3)	H55C···C17	2.70
C59···O3	2.834 (3)	H55C···O7	2.22
C60···O14^viii^	3.273 (3)	H56A···O1^i^	2.80
C8···H15A	2.75 (4)	H56A···H60A^xi^	2.53
C8···H6^v^	2.93	H56B···C24	3.08
C17···H15B^vi^	2.98 (3)	H56B···H6	2.30
C17···H55C	2.70	H56B···C41	2.97
C22···H39^vii^	3.04	H56B···H15A^i^	2.59
C23···H48	2.86	H56C···C53	2.67
C24···H27	2.64	H56C···O11	2.18
C24···H56B	3.08	H58A···O3	2.67
C26···H36^vii^	2.97	H58B···H13A^iv^	2.51
C27···H62C^ii^	3.03	H58B···O13^iv^	2.89
C27···H26^ii^	3.03	H58B···C39^iv^	2.91
C29···H2^iii^	2.94	H58B···O3	2.57
C29···H13B	2.93 (3)	H59A···C41	2.74
C30···H27	3.01	H59A···O3	2.25
C31···H43	3.07	H59B···H2	2.17
C31···H11	3.03	H59B···H13B^iii^	2.49
C32···H48	2.64	H59B···O2^iii^	2.83
C33···H49	2.87	H59C···O15^i^	2.89
C35···H61B^ix^	3.09	H59C···H47B^xiii^	2.50
C35···H54^ii^	3.04	H60A···O14^viii^	2.91
C36···H26^ii^	2.88	H60A···O11	2.64
C36···H60B^ix^	3.07	H60A···H56A^xi^	2.53
C37···H49	2.64	H60A···C56^xi^	3.06
C38···H4^i^	2.88	H60B···O14^viii^	2.75
C38···H14A^vii^	2.79 (4)	H60B···C36^viii^	3.07
C38···H511^x^	3.03	H60B···O11	2.58
C39···H58B^vi^	2.91	H60B···H14B^viii^	2.52
C39···H63B^vi^	3.03	H61A···H55A^vii^	2.55
C41···H59A	2.74	H61B···C35^viii^	3.09
C41···H13A^iv^	2.96 (3)	H62B···C42^vi^	3.09
C41···H62C^ii^	3.04	H62C···C27^vii^	3.03
C41···H56B	2.97	H62C···H27^vii^	2.55
C42···H62B^iv^	3.09	H62C···C41^vii^	3.04
C42···H26	3.01	H63B···C39^iv^	3.03
C43···H44	3.07	H63C···C49	3.04
C44···H47A^iv^	2.97	H63C···H49	2.59
C44···H43	3.08	H511···C38^x^	3.03
C47···H15B^vi^	2.96 (3)	H511···O1^vii^	2.47
C49···H63C	3.04	H511···H1^vii^	2.43
			
C41—O4—C58	116.7 (2)	C39—C31—C57	119.6 (2)
C35—O1—H1	109	O5—C31—C57	118.1 (2)
C17—O8—C47	116.56 (18)	C17—C32—C23	121.7 (2)
C31—O5—H5	109	C17—C32—C34	118.1 (2)
C53—O12—C60	116.77 (18)	C23—C32—C34	120.0 (2)
C42—O9—H9A	109	C14—C34—C32	113.26 (19)
H13A—O13—H13B	118 (3)	N3—C34—C32	108.97 (18)
H14A—O14—H14B	103 (4)	N3—C34—C14	110.25 (18)
C9—N1—C29	126.0 (2)	C31—C39—C48	120.0 (2)
C29—N2—C30	123.7 (2)	O8—C47—C62	108.1 (2)
C29—N1—H11	117	C14—C48—C39	121.1 (2)
C9—N1—H11	117	C26—C57—C31	120.0 (2)
C29—N2—H2	118	C14—C26—H26	119
C30—N2—H2	118	C57—C26—H26	119
H15A—O15—H15B	115 (3)	C14—C34—H34	108
C8—N3—C34	124.5 (2)	C32—C34—H34	108
C8—N4—C23	124.4 (2)	N3—C34—H34	108
C34—N3—H3	118	C31—C39—H39	120
C8—N3—H3	118	C48—C39—H39	120
C23—N4—H4	118	O8—C47—H47A	110
C8—N4—H4	118	H47A—C47—H47B	108
C38—N5—C52	125.1 (2)	C62—C47—H47A	110
C33—N6—C38	124.2 (2)	C62—C47—H47B	110
C52—N5—H511	117	O8—C47—H47B	110
C38—N5—H511	117	C14—C48—H48	119
C38—N6—H6	118	C39—C48—H48	119
C33—N6—H6	118	H55A—C55—H55C	109
C18—C9—C24	114.0 (2)	H55A—C55—H55B	109
N1—C9—C18	110.38 (18)	C23—C55—H55A	109
N1—C9—C24	109.13 (19)	C23—C55—H55B	109
C9—C18—C27	122.5 (2)	H55B—C55—H55C	109
C27—C18—C43	117.2 (2)	C23—C55—H55C	109
C9—C18—C43	120.3 (2)	C26—C57—H57	120
C9—C24—C41	117.6 (2)	C31—C57—H57	120
C30—C24—C41	121.3 (2)	C47—C62—H62A	109
C9—C24—C30	121.0 (2)	C47—C62—H62B	109
C18—C27—C36	121.7 (2)	H62A—C62—H62C	109
O2—C29—N2	120.5 (2)	H62A—C62—H62B	109
N1—C29—N2	116.4 (2)	H62B—C62—H62C	109
O2—C29—N1	123.2 (2)	C47—C62—H62C	109
C24—C30—C59	127.5 (2)	C42—C22—C54	119.7 (3)
N2—C30—C24	119.3 (2)	C52—C25—C54	119.9 (2)
N2—C30—C59	113.2 (2)	C49—C25—C52	122.4 (2)
C36—C35—C51	119.2 (2)	C49—C25—C54	117.6 (2)
O1—C35—C36	122.1 (3)	N6—C33—C37	118.7 (2)
O1—C35—C51	118.7 (2)	C37—C33—C56	129.0 (2)
C27—C36—C35	120.1 (2)	N6—C33—C56	112.3 (2)
O4—C41—C24	111.2 (2)	C33—C37—C52	120.4 (2)
O3—C41—O4	121.9 (2)	C33—C37—C53	122.1 (2)
O3—C41—C24	126.9 (3)	C52—C37—C53	117.3 (2)
C18—C43—C51	121.6 (2)	O10—C38—N5	123.8 (2)
C35—C51—C43	120.2 (2)	N5—C38—N6	115.6 (2)
O4—C58—C63	108.7 (2)	O10—C38—N6	120.6 (2)
N1—C9—H9	108	O9—C42—C22	118.0 (2)
C24—C9—H9	108	O9—C42—C44	122.1 (3)
C18—C9—H9	108	C22—C42—C44	119.9 (2)
C36—C27—H27	119	C42—C44—C49	119.8 (2)
C18—C27—H27	119	C25—C49—C44	121.5 (2)
C27—C36—H36	120	N5—C52—C37	109.2 (2)
C35—C36—H36	120	N5—C52—C25	110.05 (19)
C18—C43—H43	119	C25—C52—C37	112.7 (2)
C51—C43—H43	119	O11—C53—O12	121.8 (2)
C35—C51—H51	120	O11—C53—C37	126.9 (3)
C43—C51—H51	120	O12—C53—C37	111.3 (2)
C63—C58—H58A	110	C22—C54—C25	121.5 (2)
O4—C58—H58B	110	O12—C60—C61	108.6 (2)
C63—C58—H58B	110	C42—C22—H22	120
O4—C58—H58A	110	C54—C22—H22	120
H58A—C58—H58B	108	C42—C44—H44	120
H59A—C59—H59B	109	C49—C44—H44	120
C30—C59—H59C	109	C25—C49—H49	119
H59A—C59—H59C	109	C44—C49—H49	119
H59B—C59—H59C	109	C37—C52—H52	108
C30—C59—H59B	110	N5—C52—H52	108
C30—C59—H59A	109	C25—C52—H52	108
C58—C63—H63A	109	C22—C54—H54	119
C58—C63—H63C	109	C25—C54—H54	119
C58—C63—H63B	110	C33—C56—H56A	109
H63B—C63—H63C	109	C33—C56—H56B	109
H63A—C63—H63C	109	H56B—C56—H56C	109
H63A—C63—H63B	109	H56A—C56—H56C	109
O6—C8—N4	120.5 (2)	C33—C56—H56C	109
O6—C8—N3	123.8 (2)	H56A—C56—H56B	109
N3—C8—N4	115.7 (2)	O12—C60—H60A	110
C34—C14—C48	122.4 (2)	O12—C60—H60B	110
C26—C14—C34	119.9 (2)	C61—C60—H60A	110
C26—C14—C48	117.7 (2)	C61—C60—H60B	110
O7—C17—O8	121.6 (2)	H60A—C60—H60B	108
O7—C17—C32	126.6 (2)	C60—C61—H61C	109
O8—C17—C32	111.9 (2)	H61A—C61—H61C	110
N4—C23—C55	112.9 (2)	H61B—C61—H61C	109
N4—C23—C32	118.6 (2)	H61A—C61—H61B	109
C32—C23—C55	128.5 (2)	C60—C61—H61A	109
C14—C26—C57	121.6 (2)	C60—C61—H61B	109
O5—C31—C39	122.3 (2)		
			
C58—O4—C41—O3	5.0 (4)	C36—C35—C51—C43	0.6 (4)
C58—O4—C41—C24	−175.9 (2)	C51—C35—C36—C27	−0.4 (4)
C41—O4—C58—C63	173.1 (2)	C18—C43—C51—C35	−0.7 (4)
C47—O8—C17—O7	2.6 (4)	C48—C14—C26—C57	−1.7 (3)
C47—O8—C17—C32	−177.5 (2)	C34—C14—C26—C57	−179.5 (2)
C17—O8—C47—C62	173.3 (2)	C26—C14—C48—C39	1.5 (3)
C60—O12—C53—O11	3.7 (4)	C34—C14—C48—C39	179.3 (2)
C60—O12—C53—C37	−174.8 (2)	C26—C14—C34—N3	85.8 (3)
C53—O12—C60—C61	175.9 (2)	C26—C14—C34—C32	−151.9 (2)
C29—N1—C9—C18	104.2 (3)	C48—C14—C34—N3	−92.0 (3)
C9—N1—C29—O2	−173.0 (2)	C48—C14—C34—C32	30.4 (3)
C29—N1—C9—C24	−21.8 (3)	O7—C17—C32—C34	−172.9 (3)
C9—N1—C29—N2	8.1 (4)	O8—C17—C32—C23	−178.5 (2)
C29—N2—C30—C59	165.2 (2)	O7—C17—C32—C23	1.4 (4)
C30—N2—C29—N1	11.2 (3)	O8—C17—C32—C34	7.2 (3)
C29—N2—C30—C24	−13.1 (4)	C55—C23—C32—C17	1.0 (4)
C30—N2—C29—O2	−167.8 (2)	N4—C23—C32—C17	−178.9 (2)
C8—N3—C34—C32	−31.1 (3)	N4—C23—C32—C34	−4.7 (4)
C34—N3—C8—O6	−166.1 (2)	C55—C23—C32—C34	175.2 (2)
C34—N3—C8—N4	15.6 (3)	C14—C26—C57—C31	0.1 (4)
C8—N3—C34—C14	93.8 (3)	O5—C31—C39—C48	179.4 (2)
C23—N4—C8—N3	9.5 (3)	C39—C31—C57—C26	1.7 (4)
C8—N4—C23—C32	−14.6 (4)	C57—C31—C39—C48	−1.9 (3)
C8—N4—C23—C55	165.5 (2)	O5—C31—C57—C26	−179.6 (2)
C23—N4—C8—O6	−168.8 (2)	C17—C32—C34—C14	75.7 (3)
C38—N5—C52—C37	−28.1 (3)	C23—C32—C34—N3	24.4 (3)
C38—N5—C52—C25	96.2 (3)	C23—C32—C34—C14	−98.7 (3)
C52—N5—C38—O10	−168.4 (2)	C17—C32—C34—N3	−161.2 (2)
C52—N5—C38—N6	12.9 (3)	C31—C39—C48—C14	0.3 (3)
C38—N6—C33—C56	165.4 (2)	C54—C22—C42—O9	−179.5 (2)
C33—N6—C38—O10	−168.1 (2)	C54—C22—C42—C44	2.0 (4)
C33—N6—C38—N5	10.7 (3)	C42—C22—C54—C25	−1.8 (4)
C38—N6—C33—C37	−14.9 (4)	C52—C25—C49—C44	178.3 (2)
N1—C9—C24—C41	−165.2 (2)	C54—C25—C49—C44	0.6 (3)
C18—C9—C24—C30	−105.1 (3)	C49—C25—C52—N5	−90.3 (3)
C18—C9—C24—C41	70.9 (3)	C49—C25—C52—C37	31.9 (3)
C24—C9—C18—C27	28.6 (3)	C54—C25—C52—N5	87.3 (3)
N1—C9—C18—C27	−94.6 (3)	C54—C25—C52—C37	−150.5 (2)
N1—C9—C18—C43	85.2 (3)	C49—C25—C54—C22	0.5 (3)
C24—C9—C18—C43	−151.6 (2)	C52—C25—C54—C22	−177.3 (2)
N1—C9—C24—C30	18.8 (3)	N6—C33—C37—C52	−3.9 (4)
C9—C18—C27—C36	179.5 (2)	N6—C33—C37—C53	−179.2 (2)
C9—C18—C43—C51	−179.3 (2)	C56—C33—C37—C52	175.8 (2)
C43—C18—C27—C36	−0.3 (3)	C56—C33—C37—C53	0.5 (4)
C27—C18—C43—C51	0.5 (3)	C33—C37—C52—N5	22.4 (3)
C9—C24—C41—O3	−172.6 (3)	C33—C37—C52—C25	−100.2 (3)
C9—C24—C41—O4	8.3 (3)	C53—C37—C52—N5	−162.1 (2)
C41—C24—C30—N2	−179.6 (2)	C53—C37—C52—C25	75.3 (3)
C41—C24—C30—C59	2.3 (4)	C33—C37—C53—O11	3.9 (4)
C9—C24—C30—C59	178.2 (2)	C33—C37—C53—O12	−177.6 (2)
C9—C24—C30—N2	−3.8 (4)	C52—C37—C53—O11	−171.5 (3)
C30—C24—C41—O4	−175.7 (2)	C52—C37—C53—O12	6.9 (3)
C30—C24—C41—O3	3.4 (4)	O9—C42—C44—C49	−179.5 (2)
C18—C27—C36—C35	0.2 (4)	C22—C42—C44—C49	−1.0 (4)
O1—C35—C51—C43	179.3 (2)	C42—C44—C49—C25	−0.3 (4)
O1—C35—C36—C27	−179.1 (2)		

Symmetry codes: (i) *x*, −*y*+1/2, *z*+1/2; (ii) −*x*+1, *y*+1/2, −*z*+1/2; (iii) −*x*+1, −*y*+1, −*z*+1; (iv) *x*−1, *y*, *z*; (v) *x*, −*y*+1/2, *z*−1/2; (vi) *x*+1, *y*, *z*; (vii) −*x*+1, *y*−1/2, −*z*+1/2; (viii) −*x*, *y*−1/2, −*z*+1/2; (ix) −*x*, *y*+1/2, −*z*+1/2; (x) −*x*+1, −*y*, −*z*+1; (xi) −*x*, −*y*, −*z*+1; (xii) *x*+1, −*y*+1/2, *z*−1/2; (xiii) *x*−1, −*y*+1/2, *z*+1/2.

### Hydrogen-bond geometry (Å, °)

**Table:**

*D*—H···*A*	*D*—H	H···*A*	*D*···*A*	*D*—H···*A*
N1—H11···O5	0.86	2.14	3.002 (3)	176
N2—H2···O2^iii^	0.86	2.05	2.882 (3)	162
N3—H3···O9	0.86	2.25	3.079 (3)	161
N4—H4···O10^v^	0.86	1.99	2.854 (3)	177
N5—H511···O1^vii^	0.86	2.47	3.278 (3)	158
N6—H6···O6^i^	0.86	2.05	2.862 (3)	157
O1—H1···O14	0.82	1.84	2.657 (3)	171
O5—H5···O13	0.82	1.82	2.633 (3)	172
O9—H9A···O15	0.82	1.81	2.627 (3)	174
O13—H13A···O3^vi^	0.89 (3)	1.91 (3)	2.789 (3)	172 (3)
O13—H13B···O2	0.86 (3)	2.02 (3)	2.834 (3)	158 (3)
O14—H14A···O10^ii^	0.93 (4)	1.88 (4)	2.752 (3)	156 (4)
O14—H14B···O11^ix^	0.86 (3)	2.02 (3)	2.856 (3)	164 (3)
O15—H15A···O6	1.03 (4)	1.78 (4)	2.747 (3)	155 (3)
O15—H15B···O7^iv^	0.88 (3)	1.93 (3)	2.809 (3)	173 (3)

Symmetry codes: (iii) −*x*+1, −*y*+1, −*z*+1; (v) *x*, −*y*+1/2, *z*−1/2; (vii) −*x*+1, *y*−1/2, −*z*+1/2; (i) *x*, −*y*+1/2, *z*+1/2; (vi) *x*+1, *y*, *z*; (ii) −*x*+1, *y*+1/2, −*z*+1/2; (ix) −*x*, *y*+1/2, −*z*+1/2; (iv) *x*−1, *y*, *z*.
